# Introduction of organised mammography screening in Tyrol: results following first year of complete rollout

**DOI:** 10.1186/1471-2458-11-673

**Published:** 2011-08-30

**Authors:** Willi Oberaigner, Martin Daniaux, Sabine Geiger-Gritsch, Rudolf Knapp, Uwe Siebert, Wolfgang Buchberger

**Affiliations:** 1 Department of Clinical Epidemiology of the Tyrolean State Hospitals Ltd., Cancer Registry of Tyrol, Innsbruck, Austria; 2 Institute of Public Health, Medical Decision Making and Health Technology Assessment, Department of Public Health and Health Technology Assessment, UMIT - University for Health Sciences, Medical Informatics and Technology, Hall i.T., Austria; 3 ONCOTYROL - Center for Personalized Cancer Medicine, Innsbruck, Austria; 4 Innsbruck Medical University, Department of Radiology, Innsbruck, Austria; 5 Kufstein County Hospital, Department of Radiology, Kufstein, Austria; 6 Center for Health Decision Science, Department of Health Policy and Management, Harvard School of Public Health, Boston, MA, USA; 7 Institute for Technology Assessment and Department of Radiology, Massachusetts General Hospital, Harvard Medical School, Boston, MA, USA; 8 Tyrolean State Hospitals Ltd., Medical Department, Innsbruck, Austria

## Abstract

**Background:**

In Tyrol, Austria, the existing system of spontaneous mammography screening was switched in 2007 to an organised program by smoothly changing the established framework. This process followed most EU recommendations for organised mammography screening with the following exceptions: women aged 40-49 are part of the target population, screening is offered annually to the age group 40-59, breast ultrasound is available as an additional diagnostic tool, and double reading has not yet been implemented. After a pilot phase the program was rolled out to all of Tyrol in June 2008. The aim of this study was to analyse the performance of the organised screening system by comparing quality indices and recommended levels given in the well-established EU guidelines.

**Methods:**

Working from the results of the pilot phase, we extended the organised mammography system to all counties in Tyrol. All women living in Tyrol and covered by compulsory social insurance were invited for a mammography, in the age group 40-59 annually and in the age group 60-69 biennially. Screening mammography was offered mainly by radiologists in private practice, with further assessment performed at hospitals. Using the screening database, all well-established performance indicators were analysed and compared with accepted/desired levels as per the EU guidelines.

**Results:**

From June 2008 to May 2009, 120,440 women were invited. Per 1000 mammograms, 14 women were recalled for further assessment, nine underwent biopsy and four cancer cases were detected. Of invasive breast cancer cases, 32.3% and 68.4% were ≤ 10 mm and ≤ 15 mm in size, respectively, and 79.2% were node-negative. The positive predictive value for further assessment and for biopsy was 25.9% and 39.9%, respectively. Estimated two-year participation rate was 57.0%. In total, 14 interval cancer cases were detected during one year of follow-up; this is 18.4% of the background incidence rate.

**Conclusions:**

In Tyrol, Austria, an organised mammography screening program was implemented in a smooth transition from an existing spontaneous screening system and was completely rolled out within a short time. The high level of performance already seen in the pilot phase was maintained after rollout, and improvements resulting from the pilot phase were affirmed after one year of complete rollout.

## Background

Breast cancer is the leading cause of female cancer death in all industrialised countries (and also worldwide), and the breast is also the leading incident cancer site for females [1]. Therefore, screening methods for breast cancer are of greatest public health importance. A recently published Cochrane Review, which assessed the effect of mammography screening for breast cancer on mortality and morbidity concluded that screening is likely to reduce breast cancer mortality [2].

In 2006, in Tyrol, Austria, the decision was made to change the existing spontaneous mammography screening system to an organised program while, on the one hand, making best possible use of the mammography screening network established over the previous fifteen years and, on the other hand, following most EU recommendations for organised mammography screening. After a pilot phase conducted in two central counties of Tyrol covering 40% of the population from June 2007 to May 2008 [3], the organised system was completely rolled out to all of Tyrol in June 2008. It was possible to establish a country-wide mammography screening program in a very short time, which differs only in the following aspects from the EU guidelines [4]: women aged 40-49 are part of the target population, screening is offered annually in the age group 40-59, breast ultrasound is available as an additional diagnostic tool, and double reading has not yet been implemented.

To our knowledge, some European countries still have no organised mammography screening program or are in the process of planning to set up such a system [5,6]. Therefore, the Tyrolean experience can make an important contribution to deciding how to switch a health system with spontaneous mammography screening to an organised screening program that meets well-accepted quality guidelines.

It was the aim of this study to analyse the performance of the organised mammography screening system after complete rollout to all counties in Tyrol by measuring the quality indicators recommended by the EU guidelines [4] and to determine whether the high quality observed in the pilot phase could be affirmed after rollout.

## Methods

### Study population, invitation

**Figure 1:**
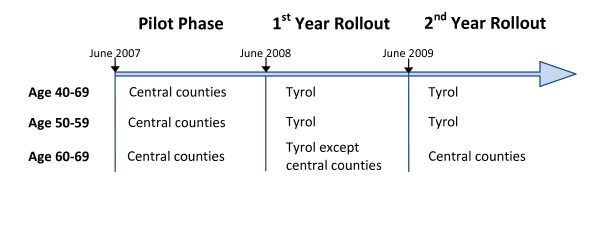
**Mammography screening system Tyrol, invitation scheme**.

The target population in the first year of complete rollout from June 2008 to May 2009 included all women aged 40 to 69 living in Tyrol and covered by compulsory social insurance, which is more than 97% of the population (personal communication). The main health insurance carrier sent out personal invitation letters to all the women in the target population in the month in which the women had their birthday: women aged 40-59 annually, and women aged 60-69 biennially. As women aged 60-69 and living in the two central counties of Tyrol where the pilot phase was conducted had already been invited in the pilot year, this group of women was not invited again in the first year of rollout (Figure 1). Mammography screening was offered by 22 screening units; thirteen of them were run by radiologists in private practice and nine by hospital outpatient departments. The mammogram was read by only one radiologist; ultrasound (US) was offered to women at the radiologist's discretion. Assessment was offered by nine hospital radiology units in the study area and included clinical inspection, mammography, US, magnetic resonance imaging (MRI) and biopsy as needed. Women were recalled for assessment either directly by the screening unit or by the general practitioner. The one large assessment unit at Innsbruck Medical University Hospital works closely with a breast cancer centre that was EUSOMA-certified in March 2010 [7]. All radiologists participating in the program underwent training and received ÖRG (Austrian Radiology Association) certification. In the median, private radiologists and hospital units performed 3234 and 1639 mammograms per year, respectively. The mammography screening system has been described in more detail elsewhere [3].

### Data collection

All mammography units registered basic information in a database. Screening and assessment information was transferred to a central database after pseudonymising the woman's social insurance number [3]. In addition, data on tumour characteristics were collected by the Cancer Registry of Tyrol.

### Statistical analysis

The screening and assessment data were realised as STATA datasets. Linkage between screening data, assessment data and Cancer Registry data is based on the pseudonym number. We reported numbers and proportions as defined in the EU guidelines [4]. For some indices, population-based rates were computed using the official population data supplied by Statistics Austria. No statistical testing was applied. All reporting was done with STATA Version 11 [8].

Performance indicators were reported from all screens in women aged 40-69 between June 2008 and May 2009.

Participation rate was calculated following a cohort approach: we counted every woman only once in the observation period, which was either one year or two years. Due to the fact that nearly half of women aged 40 to 59, who attend screening regularly, do not return for screening in the first year although they are invited annually, we computed for that age group also a two-year participation rate, meaning an observation period of two years.

Data on all mammography investigations performed in Tyrol are transferred to the screening database. A small portion (5.9%) of the women refused consent for data transfer to the screening database and we therefore receive only an empty dataset. Of all other mammography data, 76% belong to the screening population. By assuming this same proportion of 76% for the empty dataset, we calculated a proportion of 4.5% to be added to the observed participation rate accounting for empty datasets describing real numbers of mammography screening investigations.

As spontaneous mammography screening was already introduced to Tyrol in the early 1990s, the underlying background incidence rate (BIR) was defined by years of diagnosis 1988-1990.

This study was conducted in conformity with the Helsinki Declaration [9]. The project was approved by the Ethics Committee of Innsbruck Medical University.

## Results

From June 2008 to May 2009 120,440 women in the target population were invited; this excluded women aged 60-69 and living in the two central counties where the pilot phase was conducted, who fell in the biennial screening interval for that age group and were thus not invited again in this first year of complete rollout (Figure 1). The observed one- and two-year participation rates were 31.6% and 52.5%, respectively (Table 1). Participation was higher in younger women. For example, the two-year observed participation rate was 55.1% in women aged 40-49 versus 50.3% in women aged 50-69.

**Table 1:** Invitation system: Number of women invited and participation rates

	40-49	50-69	Total (40-69)
Women invited	56,888	63,552	120,440
Observed one-year participation rate	32.6%	30.6%	31.6%
Estimated one-year participation rate	37.1%	35.1%	36.1%
Observed two-year participation rate	55.1%	50.3%	52.5%
Estimated two-year participation rate	59.6%	54.8%	57.0%

Performance indicators were analysed for all screens performed in the first year of rollout, namely 42,834 screens. Of the women 75.5% underwent additional US (80.9% in women aged 40-49). Breast density (ACR 3/4) was the reason for additional US in 52.7% and 39.6% of women aged 40-49 and 50-69, respectively (Table 2).

**Table 2:** Additional ultrasound at screening

	40-49	50-69	Total
Ultrasound following mammography screening	15,126 (80.9%)	17,196 (71.2%)	32,322 (75.5%)
Reason for ultrasound:			
Breast density (ACR 3/4)	7,971 (52.7%)	6,806 (39.6%)	14,777 (45.7%)
Equivocal finding	1,801 (11.9%)	2,318 (13.5%)	4,119 (12.7%)
Other	5,354 (35.4%)	8,072 (46.9%)	13,426 (41.5%)

Per 1000 screens, 14 women were recalled for further assessment. Screening result was unknown for a total of 98 cases (0.2% of screens). Per 1000 screens, nine underwent biopsy. Of all biopsies, 86% were core biopsies and 3% open biopsies (13 cases). We observed 3.6 screen-detected cancers per 1000 screens or a total of 153 breast cancer cases, of which 9.2% were diagnosed as in situ cancers. The positive predictive value (PPV) was 25.9% for further assessment, 39.9% for total biopsy and 45.8% for core biopsy. PPV was lower in age group 40-49 (18.7%, 31.3% and 34.9% for further assessment, total biopsy and core biopsy, respectively). Performance parameters are summarised in Table 3.

**Table 3:** Performance parameters

	40-49^1)^	50-69^1)^	Total^1)^
Recall for further assessment rate [per 1000 screens] and number of recalls^2)^	14.6 (273)	13.2 (318)	13.9 (591)
Intermediate screening test recommended in six months	18.9 (354)	12.1 (292)	15.1 (646)
Screening result unknown^3)^	2.8 (52)	1.9 (46)	2.3 (98)
**Biopsy rate [per 1000 screens]**	8.7 (163)	9.1 (220)	8.9 (383)
**Cancer detection rate [per 1000 screens]**	2.7 (51)	4.2 (102)	3.6 (153)
Invasive	2.5 (46)	3.9 (93)	3.3 (139)
In situ	0.3 (5)	0.4 (9)	0.3 (14)
**Proportion of in situ cases**	9.8%	8.8%	9.2%
**Ratio screening breast cancer detection rate vs. background incidence rate^4)^**	2.1	2.0	2.0
**PPV assessment**	18.7% (51/273)	32.1% (102/318)	25.9% (153/591)
**PPV biopsy**	31.3% (51/163)	46.4% (102/220)	39.9% (153/383)

^1) ^Rates were rounded to one decimal; numbers in brackets are numbers of cases (i.e. recall rate, biopsy rate, cancer detection rate). PPV is rounded to one decimal; numbers in brackets are detailed numbers for computing PPV.

^2) ^For one case assessment was recommended, but performed at an institution outside the screening system.

^3) ^Cases with BI-RADS 0 without assessment were treated as unknown.

^4) ^Background incidence rate defined by years of diagnosis 1988-1992.

Of 139 invasive cancers diagnosed in screening, four changed to "in situ cancer" after final diagnosis. Two invasive cancer cases did not undergo surgery because of metastatic disease. Finally, three invasive cancer cases underwent neoadjuvant therapy and it was not possible to identify preoperative staging.

Of all invasive cancers detected and finally proven, 32.3% and 68.4% showed tumour size ≤10 mm and ≤15 mm, respectively. Lymph node involvement was observed in 20.8% of invasive cancer cases (Table 4).

For invasive cancers, 90.6% of further assessments were carried out within five working days after screening and 87.1% and 90.1% underwent surgery within ten days and 15 days after decision to operate, respectively. For all cases except invasive cancers, 73.7% underwent assessment within five working days and 17.1% after ten or more working days (Table 5).

We observed a total of 14 interval cancer cases within one year after screening in all of Tyrol, five in age group 40-49, giving an interval cancer rate of 20.8% and 17.8% of the background incidence rate for age groups 40-49 and 50-69, respectively (Table 6). Table 7 shows the results for the most important quality indicators of the EU guidelines [4] restricted to age group 50-69. Given that the organised system was introduced after more than a decade of spontaneous mammography screening in Tyrol, as reference values we chose the accepted and desired ranges of EU quality indicators for subsequent rounds. Most of the indicators were within the EU range, except the participation rate (54.8% vs. the limit of 75%), the proportion of II+ cancers (33.3% vs. the limit of 25%), the proportion of invasive cancers (91.2%, this is slightly above the limit of 90%) and the proportion of cases that underwent surgery within ≤ 15 working days after decision to operate (87.3%, this is slightly below the limit of 90%).

**Table 4:** Characteristics of invasive cancer cases

	40-49	50-69	Total
**Tumour size (mm): N = 133**	13; 4-25	12; 1-35	13; 1-35
Median; range			
Tumour size (mm):			
< = 10 mm	14 (32.6%)	29 (32.2%)	43 (32.3%)
< = 15 mm	28 (65.1%)	63 (70.0%)	91 (68.4%)
11-20 mm^1)^	23 (53.5%)	42 (46.7%)	65 (48.9%)
> 20 mm	6 (14.0%)	19 (21.1%)	25 (18.8%)
			
**Lymph node involvement**	8 (18.6%)	19 (21.8%)^1)^	27 (20.8%)
**Staging according to UICC**			
I	30 (69.8%)	55 (61.8%)	85 (64.4%)
II	13 (30.2%)	31 (34.8%)	44 (33.3%)
III		3 (3.4%)	3 (2.3%)

**Notes: **Of 139 invasive cancer cases, four cases were finally "in situ"; two invasive cancer cases did not undergo surgery because of metastatic status. Three cases were without lymph node status because of neoadjuvant therapy and because we could not identify pretherapeutic TNM stage.

**Table 5:** Waiting times

Invasive cancers			
	40-49	50-69	Total
Screening to assessment			
≤ 5 wd	40 (87.0%)	86 (92.5%)	126 (90.6%)
6-10 wd	3 (6.5%)	3 (3.2%)	6 (4.3%)
> 10 wd	3 (6.5%)	4 (4.3%)	7 (5.0%)
Decision to operate to date of therapy			
≤ 10 wd	42 (93.3%)	73 (83.9%)	115 (87.1%)
11-15 wd	1 (2.2%)	3 (3.4%)	4 (3.0%)
16-30 wd	1 (2.2%)	4 (4.6%)	5 (3.8%)
> 30 wd	1 (2.2%)	7 (8.0%)	8 (6.1%)
**All screens except those ending in invasive cancers**			
	**40-49**	**50-69**	**Total**

Screening to assessment			
≤ 5 wd	162 (72.3%)	166 (75.1%)	328 (73.7%)
6-10 wd	17 (7.6%)	24 (10.9%)	41 (9.2%)
> 10 wd	45 (20.1%)	31 (14.0%)	76 (17.1%)

wd: working days

**Table 6:** Interval cancer rate within first year

	40-49	50-69	Total
Interval cancer rate per 100,000 screens (number of cases in brackets)	26.7 (5)	37.3 (9)	32.7 (14)
Proportion of background incidence rate^1) ^(in percent)	20.8%	17.8%	18.4%

^1) ^based on years of diagnosis 1988-1990

**Table 7:** EU Guidelines, quality indicators (with accepted and desired levels)

	Tyrol 50-69	EU-accepted^1)^	EU-desired^1)^
Participation rate (after two years of observation)	54.8%	> 70	> 75
Recall for further assessment rate^1)^	1.3 (318)	< 5%	< 3%
Breast cancer detection rate	2.0 * BIR	1.5*BIR	> 1.5*BIR
Interval cancer rate/Background incidence rate (BIR) 0-11 months	18% (9)	30%	< 30%
Proportion of screen-detected cancers that were invasive	91.2% (93/102)	90%	80%-90%
Proportion of screen-detected cancers that were stage II+	33.3% (34/102)^2)^	25	< 25%
Node-negative cancer/Total invasive cancers screen-detected	78.2%	75%	> 75%
Invasive cancers ≤ 10 mm/Total invasive cancers	32.2%	≥25%	≥30%
Proportion of invasive cancers that were ≤ 15 mm in size	70.0%	50%	> 50%
Time between results of screening and assessment < = 5 wd^3)^	92.5%	90%	> 90%
Time between decision to operate and surgery < = 15 wd^4)^	87.3%	90%	> 90%

^1) ^We took reference values for subsequent rounds, because we had a decade of spontaneous screening before beginning the organised program.

^2) ^One of 150 cases without UICC staging (no staging because of neoadjuvant therapy).

^3) ^We show time between screening and performed assessment, not offered assessment.

^4) ^We show time between decision to operate and surgery performed (not between decision to operate and date offered for surgery).

## Discussion

We analysed performance after one year of rolling out an organised mammography screening program to all counties in Tyrol. The organised program was established in a smooth transition from an existing spontaneous mammography screening system, instead of setting up a completely new screening system, and was previously tested in a pilot phase comprising 40% of the target population [3]. Although not all EU recommendations were followed, most quality indicators are in the range of accepted/desired levels given by the EU guidelines [4]. The only parameter that clearly missed the EU guidelines was the participation rate: the two-year participation rate was 57% as compared to the 75% recommended by the EU guidelines. In our opinion, a cumulative participation rate of 57% after two years of observation looks successful when compared to neighbouring countries [10-12]. Nevertheless, it is not the goal we aimed for.

The **strengths **of the Tyrolean breast cancer screening program are its implementation and performance: we were able to set up an organised population-based screening program within a short time with minimal additional resources that shows good performance. In summary, the recall for further assessment rate and the biopsy rate are fairly low, PPV was good as compared to other programs, only few open biopsies were performed, and despite the lack of double reading the interval cancer rate of 20% of the underlying BIR is rather good as compared to other programs [10,13-15].

However, this study has **several weaknesses**. First, up to now we have not implemented double reading as recommended in the EU guidelines. Interestingly, performance parameters and especially interval cancer rate showed that also without double reading an acceptable quality level was achieved. One reason could be the extensive use of additional US, about three of four women underwent additional US. The real benefit of US in a population-based mammography screening program is currently under discussion and has to be further evaluated [16,17]. Calculation of the interval cancer rate is reliant on the completeness of the Cancer Registry of Tyrol, which covers the target population. Completeness of incidence data in general has been shown to be very good [3,18]. In order to be able to analyse interval cancer rates for the screening program the timeliness of registration of breast cancer was improved, and linkage between cancer registry data and screening data is based on pseudonymising the social insurance number, which is read electronically. In the meantime, we have also assessed interval cancer in the time window 12 to 23 months for the pilot phase of the Tyrol program, see [3], and found five interval cancer cases in age group 40-49 (55% of BIR) and seven interval cancer cases in age group 50-69 (33% of BIR), data not shown.

Second, the average number of screens read by a radiologist in Tyrol per year (about 3200) does not meet the EU recommendation of 5000. A recent publication [19] showed that annual numbers below 5000 can still provide good sensitivity and acceptable false-positive rates.

Third, we used BI-RADS categories instead of a single yes/no rule for recall for further assessment. Some radiologists still use BI-RADS 0 (meaning unclear result) in a small number of cases (0.2% of all screens), and 15 per 1000 screens were invited to an intermediate screening test six months following a BI-RADS 3 screening result. Due to this inconsistency, the current program includes the following modifications: BI-RADS 0 is no longer allowed and BI-RADS 3 is strictly associated with recall for further assessment.

Many countries have run a mammography screening program for decades or for a shorter time. On the other hand, there are still some countries with no organised breast cancer screening program. For those countries thinking of or already in the process of introducing a mammography screening program, our manner of introducing an organised program can serve as one how-to example. In our opinion, the greatest difference between our approach and other approaches, especially compared to Germany, is the smooth transition made from an existing spontaneous program to an organised population-based screening. We made use of the network of screening and assessment units that had already been set up during spontaneous screening and added an invitation system covering the entire population of Tyrol, a screening database that allows quality indices to be monitored and a well-defined training program for both screening and assessment units. With this strategy we were able to meet most EU quality indices within a very short time.

## Conclusions

In Tyrol, Austria, an organised mammography screening system realised in a smooth transition from an existing spontaneous screening system was rolled out in a short time. The high level of performance already observed in the pilot phase has not changed after the first year of complete rollout. Improvements suggested during the pilot phase were affirmed after rollout: it will be necessary to concentrate on efforts to improve the participation rate, introduce double reading, change the rule for BI-RADS 3, and reduce the number of additional ultrasound exams.

## List Of Abbreviations

US: ultrasound, ÖRG: Austrian Radiology Association, ACR: American College of Radiology, MRI: magnetic resonance imaging, BIR: background incidence rate, PPV: posivite predictive value.

## Competing interests

The authors declare that they have no competing interests.

## Authors' contributions

WO and WB designed the study. WO performed the analysis and wrote the paper. SGG contributed to writing the paper and to critically reviewing the draft. WB, MD und RK contributed to the Discussion section, especially from the radiology point of view. US contributed to writing the paper. All authors reviewed and agreed to the final version of the manuscript.

## Pre-publication history

The pre-publication history for this paper can be accessed here:

http://www.biomedcentral.com/1471-2458/11/673/prepub

## References

[B1] Boyle P Levin B Eds World Cancer Report 2008 2008 Lyon: IARC Press

[B2] Gotzsche PC Nielsen M Screening for breast cancer with mammography Cochrane Database Syst Rev 2011 CD001877 10.1002/14651858.CD001877.pub4 21249649

[B3] Oberaigner W Buchberger W Frede T Daniaux M Knapp R Marth C Siebert U Introduction of organised mammography screening in tyrol: results of a one-year pilot phase BMC Public Health 2011 11 91 99 10.1186/1471-2458-11-91 21306614 PMC3048536

[B4] Perry N Broeders M de Wolf C (Eds.) European guidelines for quality assurance in breast cancer screening and diagnosis 2006 Luxembourg: Office for Official Publications of the European Communities 10.1093/annonc/mdm481 18024988

[B5] Karsa L Anttila A Ronco G Ponti A Malila N Arbyn M Cancer Screening in the European Union. Report on the implementation of the Council Recommendation on cancer screening. First Report 2008 Brussels: European Commission

[B6] Dowling EC Klabunde C Patnick J Ballard-Barbash R Breast and cervical cancer screening programme implementation in 16 countries J Med Screen 2010 17 139 146 10.1258/jms.2010.010033 20956724

[B7] Eusoma-Breast Unit-Certification Process http://www.eusoma.org/Engx/BreastUnits/AccreditationProcess.aspx?cont=ap_accredited accessed 09.05.2011

[B8] Stata Statistical Software: Release 11 2009 College Station, Tx, StataCorp LP

[B9] World Medical Association Declaration of Helsinki: ethical principles for medical research involvin human subjects http://www.wma.net/en/30publications/10policies/b3/index.html (acessed 7 November 2009)

[B10] Kooperationsgemeinschaft Mammographie (Ed.) Evaluationsbericht 2005-2007. Ergebnisse des Mammographie-Screening-Programms in Deutschland 2009 Köln

[B11] Bulliard JL Ducros C Jemelin C Arzel B Fioretta G Levi F Effectiveness of organised versus opportunistic mammography screening Ann Oncol 2009 20 1199 1202 10.1093/annonc/mdn770 19282467

[B12] Giordano L Giorgi D Ventura L Stefanini V Senore C Castagno R Paci E Segnan N Time trends of process and impact indicators in Italian breast screening programmes: 1998-2008 Epidemiol Prev 2010 34 27 34 21220835

[B13] Hofvind S Vacek PM Skelly J Weaver DL Geller BM Comparing screening mammography for early breast cancer detection in Vermont and Norway J Natl Cancer Inst 2008 100 1082 1091 10.1093/jnci/djn224 18664650 PMC2720695

[B14] Blanks RG Moss SM McGahan CE Quinn MJ Babb PJ Effect of NHS breast screening programme on mortality from breast cancer in England and Wales, 1990-8: comparison of observed with predicted mortality BMJ 2000 321 665 669 10.1136/bmj.321.7262.665 10987769 PMC27479

[B15] Smith-Bindman R Chu PW Miglioretti DL Sickles EA Blanks R Ballard-Barbash R Bobo JK Lee NC Wallis MG Patnick J Kerlikowske K Comparison of screening mammography in the United States and the United kingdom JAMA 2003 290 2129 2137 10.1001/jama.290.16.2129 14570948

[B16] Corsetti V Houssami N Ghirardi M Ferrari A Speziani M Bellarosa S Remida G Gasparotti C Galligioni E Ciatto S Evidence of the effect of adjunct ultrasound screening in women with mammography-negative dense breasts: Interval breast cancers at 1year follow-up Eur J Cancer 2011 47 1021 1026 10.1016/j.ejca.2010.12.002 21211962

[B17] Husby JA Espeland A Kalyanpur A Brocker C Haldorsen IS Double reading of radiological examinations in Norway Acta Radiol 2011 10.1258/ar.2011.100347 21498308

[B18] Oberaigner W Siebert U Are survival rates for Tyrol published in the Eurocare studies biased? Acta Oncol 2009 1 8 10.1080/02841860903188635 19714522

[B19] Buist DS Anderson ML Haneuse SJ Sickles EA Smith RA Carney PA Taplin SH Rosenberg RD Geller BM Onega TL Influence of annual interpretive volume on screening mammography performance in the United States Radiology 2011 259 72 84 10.1148/radiol.10101698 21343539 PMC3064821

